# Ancient DNA Assessment of Tiger Salamander Population in Yellowstone National Park

**DOI:** 10.1371/journal.pone.0032763

**Published:** 2012-03-12

**Authors:** Sarah K. McMenamin, Elizabeth A. Hadly

**Affiliations:** Department of Biology, Stanford University, Stanford, California, United States of America; University of Copenhagen, Denmark

## Abstract

Recent data indicates that blotched tiger salamanders (*Ambystoma tigrinum melanostictum*) in northern regions of Yellowstone National Park are declining due to climate-related habitat changes. In this study, we used ancient and modern mitochondrial haplotype diversity to model the effective size of this amphibian population through recent geological time and to assess past responses to climatic changes in the region. Using subfossils collected from a cave in northern Yellowstone, we analyzed >700 base pairs of mitochondrial sequence from 16 samples ranging in age from 100 to 3300 years old and found that all shared an identical haplotype. Although mitochondrial diversity was extremely low within the living population, we still were able to detect geographic subdivision within the local area. Using serial coalescent modelling with Bayesian priors from both modern and ancient genetic data we simulated a range of probable population sizes and mutation rates through time. Our simulations suggest that regional mitochondrial diversity has remained relatively constant even through climatic fluctuations of recent millennia.

## Introduction

Detailed contemporary genetic characteristics of a population can be analyzed to reveal aspects of its history. Nonetheless, the modern genetics of a population serve only as a snapshot of complex and dynamic processes averaged over time and biased by recent events. Incorporating ancient information into genetic studies can place a population into its historical context, and can reveal details of a population's history that might otherwise be lost with time [1], [2]. Especially when considering a population facing contemporary environmental threats, it can be highly informative to determine how the population responded to comparable historic environmental changes [3].

Amphibian populations are sensitive environmental indicators, tracking both aquatic and terrestrial environments, and their responses to historical environmental changes may lend insight into their prospects in rapidly changing modern ecosystems. Genetic variation in modern populations of tiger salamanders (*Ambystoma* spp.) suggest complex colonization, migration and bottleneck patterns through their histories that could be explored in greater detail by anchoring modern genetic variation in ancient variation. Nonetheless, no previous study has included assessments of ancient amphibian DNA, either in a phylogeographic or a population genetic context. To our knowledge, the oldest analyzed amphibian genetic material was extracted from museum specimens accessioned in 1920 [4]. In the current study, we used amphibian samples from the last 3000 years to study the history of an amphibian population that has likely experienced declines and bottlenecks in past millennia and centuries [5], as well as in recent decades [6].

Amphibians may be declining in northern Yellowstone. Letters from the late 1800s and reports as recent as the 1950s describe summer mass migrations of hundreds of thousands of adult *A. t. melanostictum* [7], but recent data suggest the population may be in decline due to changing climate, pond desiccation [6] and disease. The genetics of Yellowstone *Ambystoma* further exhibit signatures of population decline and bottlenecks: microsatellites show a deficit of rare alleles, an excess of heterozygosity and a notably ragged distribution of allele sizes [5]. However, the ragged distribution of allele sizes likely reflect bottleneck events older than the recent decline trends, and may reflect the initial colonization bottleneck after deglaciation ∼14,000 years before present (ybp). More recently, population bottlenecks may have been caused by specific climatic events such as the Medieval Warm Period (∼1200 ybp), long-term climatic fluctuations or by the historic practice of stocking ponds with fish that prey on salamanders [8]. More powerful methods can be used to date bottlenecks from modern genetic information. However in the absence of ancient genetic data it can be difficult to pinpoint with great precision when any particular bottleneck occurred and to which factors it may be attributed.

Lamar Cave in northern Yellowstone National Park, Wyoming, USA, is a rich wood rat (*Neotoma cinerea*) midden deposit chronicling over 3000 years of vertebrate community history [9]. Generations of wood rats collected and accumulated raptor pellets and carnivore scats containing vertebrate specimens originating within 5–7 km of the site [10], and this natural collection has been used to describe genetic, phenotypic and compositional changes in mammalian populations through time [11], [12], [13]. The blotched tiger salamander *Ambystoma tigrinum melanostictum* is the most abundant non-mammalian vertebrate present in the deposit, and Bruzgul *et al.* [14] used cervical vertebrae to characterize the demographics of the amphibian population through time, concluding that growth and development tracked contemporary climatic conditions over thousands of years.

In this study, we examined the mitochondrial genetics of this population across several localities in the local area and through recent geologic time, using serial coalescent modeling to develop a distribution of population sizes through time. Using parsimony based on our population genetic data, we aimed to disentangle the relative severities of ancient versus recent population declines and to determine the size at which this population has been maintained through environmental events. We focused on a region of the mitochondrial genome spanning the tRNA gene for threonine, a highly polymorphic non-coding intergenic spacer region, the tRNA gene for proline, and the D-loop region. The D-loop region is frequently targeted in studies using aDNA (see [15], [16]).

## Materials and Methods

### Ethics Statement

Handling of animals was conducted according to institutional and national guidelines. All work reviewed by the Stanford University Administrative Panel on Laboratory Animal Care and all protocols were approved under permit number 10284. All necessary approval was obtained for the described field studies through the Yellowstone National Park Research Permit Office under permit number YELL-2008-SCI-5638.

### Ancient sample selection and amplification

**Figure 1:**
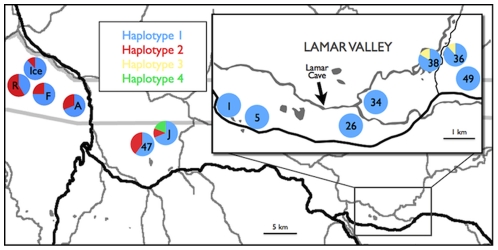
Map of field site with approximate locations of sampled ponds and proportion of individuals at each site that possessed each haplotype. Map area shows the Lamar Valley in northern Yellowstone National Park in Wyoming, USA. Thin grey lines show rivers, thick grey lines show park and state boundaries, black lines show roads. Lamar Cave site indicated with arrow. Samples collected from Lamar Cave = 16 (all Haplotype 1), samples collected from Ice Lake (Ice) = 17, Rainbow Lake (R) = 12, Pond A = 7, Pond F = 12, Pond J = 16, Pond 47 = 5, Pond 1 = 5, Pond 5 = 11, Pond 26 = 18, Pond 34 = 8, Pond 38 = 6, Pond 36 = 17, Pond 49 = 9.

Specimens of *A. t. melanostictum* were collected and sorted during excavations of Lamar Cave, a paleontological site in Yellowstone National Park (Fig. 1). The specimens were dated by stratigraphic layer, and layer age was determined through radiocarbon dating of 18 representative samples from the 16 different layers present [9]. Fifteen cervical *A. tigrinum* vertebra samples were chosen from 10 different stratigraphic layers, ranging in age from 100 to 3000 years old (Table 1). Each individual has a single cervical vertebra and the use of this bone ensured that each individual could be represented in our dataset only once.

**Table 1:** Number of samples used in this study from each stratigraphic layer in Lamar Cave, and ages and the climatic periods represented by each layer.

Layer	Number of samples used	Period	Climate	Age (years before present)
1	2	A	Little Ice Age	300
2	1			
4	1	B		600
6	2			
7	1	C	Medieval Warm Period	1200
12	1			
13	2	D		1800
14	1			
15	3	E		3300
16	3			

Period, climate and age of layers from.

We adhered to strict extraction and amplification protocols, and numerous precautions were taken to prevent contamination (see [12], [13], [17]). Neither personnel nor facilities had contact with any amphibian material prior to processing the ancient samples, and all field and lab work with modern *A. t. melanostictum* samples was initiated only after all ancient samples had been processed and analyzed. Ancient DNA (aDNA) was extracted from samples in a designated aDNA facility physically isolated from any area in which amplification or analyses were performed. Personnel were not permitted to enter the aDNA area for 24 h after contact with PCR products or facilities. Prior to extracting aDNA, all the surfaces in the aDNA facility were cleaned with 25% bleach. The room, the inside of the flow hood, and all reagents and materials were irradiated with UV light for a minimum of 12 hours. Extractions and PCR setup were performed in a sterilized Class II laminar flow hood within the sterilized aDNA facility.

Extractions of ancient material were performed in small batches of 5 or fewer samples. Weight of sampled cervical vertebrae was measured and ranged from 1 to 12 mg (average = 5.4 mg). After crushing the vertebra inside of sterilized aluminum foil using a sterilized mortar and pestle, powdered material was digested in 275 µl 0.5 M EDTA pH 8.0 with 0.5% sodium dodecyl sulfate and 100 µg/ml proteinase K for 24 h at 55°C, then purified using Qiaquick® DNA purification silica columns (Qiagen Inc., Valencia, California). We included two dummy negative controls in this process, the first control added with the samples as they were weighed and powdered, and the second added at the addition of buffer during the extraction phase. Each of these controls was used alongside the samples through the amplification process to monitor for cross-contamination. A sample of the sequences were cloned and sequenced to verify sequence fidelity (see [18]).

We designed five sets of overlapping primers, altogether 729 base pairs (bp) of the mitochondrial genome:

1F: AAACATCGATCTTGTAAGTCG 1R: ATTAAAAAAGCTCCTACGCTT (to amplify a 184 bp product); 2F: CTTAAAATCAGTAAACTGC 2R: CAAGAGGGGAGGATTTTCAC (201 bp product); 3F: CAACTCGAATTTTCTATTGC 3R: CATCCAACTATCTGCCACACG (214 bp product); 4F GTAACGTGGCAACATATTATG 4R: CTGGTTAAAATCTATGGAC (201 bp product); 5F: GAGTGCCTTACTTCCCTTG 5R: GTAATATGACTGGTACTAC (205 bp product).

Amplifications were performed in 50 µL reactions containing 1 U *AmpliTaq*® Gold polymerase (Applied Biosystems, Foster City, California), 1× *Taq* Gold buffer, 5 mM MgCl_2_, 1 mM each dNTP, 1.3 mg/ml spermidine (Sigma-Aldrich, St. Louis, Missouri) and 0.25 µM of each primer. A third control was added during PCR preparation.

Samples were transferred into the physically isolated PCR room and run on a 9700 thermocycler (Applied Biosystems, Foster City, California) for 10 min at 95°C, 45 cycles of 95°C for 30 sec, 45°C for 30 sec, 72°C for 30 sec, followed by 10 min at 72°C. Every amplification reaction included a negative control with no added DNA. We tested each of the dummy negative controls collected during the extraction process, of which there were eight in total (two for each of the four extraction batches performed). These were amplified along with the actual DNA samples, and none of the dummy negative controls ever yielded product visible on an agarose gel stained with ethidium bromide. Likewise, the negative control amplification reactions containing no DNA never yielded detectable products. Our PCRs were successful for 51% of positive (DNA-containing) reactions. We found no correlation between amplification success and fragment size or sample age. Amplified fragments were purified with the Qiaquick® PCR purification kit and sequenced in both forward and reverse directions by Genaissance Pharmaceuticals (New Haven, Connecticut; now Cogenics, Inc., Houston, Texas).

### Modern sample collection and amplification

Collection and analysis of modern individuals was performed only after all ancient analyses were completed. Live *A. t. melanostictum* were collected from 13 ponds in northern Yellowstone National Park (Fig. 1) using either dip nets or funnel traps, and between 8 and 18 individuals were collected and analyzed from each location (average = 12.4). Tail clips of 3–10 mm were collected from each individual and stored in vials containing 90–100% EtOH and refrigerated until extraction; live specimens were then released where captured. DNA was extracted using either a DNeasy® Blood and Tissue kit (Qiagen Inc., Valencia, California) or by incubating tissue with 250 µl 5% Chelex 100 beads (Sigma-Aldrich, St. Louis, Missouri) for 95°C for 1 h. Amplifications were performed in 50 µL reactions containing 1 U *Taq* polymerase (Invitrogen, Carlsbad, California), 1× PCR buffer, 2 mM MgCl_2_, 0.2 mM each dNTP and 0.25 µM of primers 1F and 5R (see sequences in previous section). PCRs were run at 95°C for 2 min, 35 cycles of 95°C for 30 sec, 55°C for 30 sec, 72°C for 1 min, followed by 5 min at 72°C. PCR samples were sequenced in both forward and reverse directions by Cogenics, Inc. (Houston, Texas, formerly Genaissance Pharmaceuticals). In order authenticate our sequences (see [18]), we randomly selected seven samples and cloned each into PCR-TOPO TA vectors according to the manufacturers protocol (Invitrogen, Carlsbad, California). Ten colonies were sequenced for each sample with M13 primers, and we verified that each corresponded to the original sequence.

### Population Analyses and Coalescent Modeling

Sequence reads were aligned with Sequencher software (version 3.1.1, Gene Codes Corporation, Ann Arbor, Michigan). Mantel tests and AMOVA, both with 9999 permutations, were performed for the modern population using GenAlEx 6.2 [19], [20]. GenAlEx was also used to calculate φ_PT_, a measure of genetic variability analogous to F_ST_. Tajima's D was calculated in Arlequin 3.0 [21]. We used Bayesian Serial SimCoal [22] to model the most likely effective population sizes by simulating haplotype frequencies through time. Bayesian Serial SimCoal is similar to SIMCOAL 1.0 [23], but allows the input of Bayesian priors into the models and incorporate both modern and ancient sampling into simulations. Several mitochondrial mutation rates have been proposed for *Ambystoma* [24], [25] and rates of substitution vary between different regions of the mitochondrial genome; we therefore explored a range of mutation rates, from 0.25% to 1.75% mutations per nucleotide per million years. We assumed a generation time of four years. Samples from Lamar Cave are known to have originated within a 7-km radius of the cave site [10]. Therefore, we modeled ponds in the Lamar Valley (Fig. 1 inset), all within 7 km of the cave, as the modern descendants of the populations represented in Lamar Cave. Using ranges of priors for population size (0–40000) and mutation rate (0.25%–1.75%), we calculated the rate at which simulations produced outcomes matching the observed number of ancient mitochondrial segregating sites (*i.e.* 0 sites) or both the observed ancient segregating sites (0 sites) as well as the number of modern segregating sites for the Lamar Valley population (*i.e.* 1 site). After running Bayesian models, we ultimately performed simulations based on fixed parameters. In these simulations, we fixed both the mutation rate and the population size, then ran 1000 independent simulations and evaluated the percentage of successful simulations. A simulation was deemed successful when it produced the appropriate numbers of segregating sites in the ancient (Ancient Data) or in both the modern and ancient populations (Ancient+Modern Data). We calculated the probability of any potential population size as the percent success in these simulations.

## Results

We sampled 143 modern and 16 ancient *A. t. melanostictum* from northern Yellowstone (Fig. 1) and amplified more than 700 bp of the mitochondrial genome spanning both coding and non-coding regions. We identified only four unique haplotypes (GenBank Accession Numbers: GQ260669–GQ260817 and GQ260834–GQ260845). Each haplotype differed from the others by a single non-coding polymorphic site. The polymorphisms distinguishing Haplotypes 2 and 3 were in the D-loop, and the haplotype distinguishing Haplotype 4 was in the intergenic spacer region. Haplotype 1 (H1) was by far the most common genotype, present at every location and representing 83% of modern samples. H1 dominated the Lamar Valley region, where five out of seven sampled pond populations were composed exclusively of H1 (Fig. 1). All 16 of the ancient individuals genotyped also represented H1.

Multiple AMOVAs of the modern genetic data indicated that the population exhibited significant isolation by distance (Table 2). A Mantel test showed R_xy_ = 0.23 (p<0.001) with distance explaining 5.1% variation between ponds. The amount of subdivision within the population was significant (φ_PT_ = 0.179, p = 0.001). We calculated Tajima's D for individual ponds, for the ponds in Lamar Valley and for all ponds; in all scenarios, there was no significant deviation from 0.

**Table 2:** AMOVAs of genetic subdivision in modern northern Yellowstone based on the four identified mitochondrial haplotypes.

	DF	SS	MS	Estimated Variation	Variation
Among Populations	12	5.003	0.417	0.027	18%
Within Populations	130	16.126	0.124	0.124	23%

DF = degrees of freedom; SS = sum squares, MS = mean squares.

**Figure 2:**
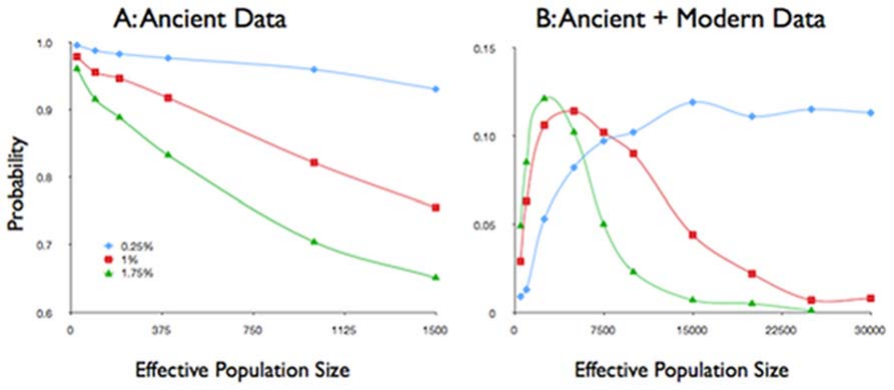
Frequencies with which number of observed segregating sites are simulated under different mutation rate scenarios. A: Results of simulating the number of segregating sites observed in ancient samples from Lamar Cave. B: Results of simulating segregating site frequency found in modern Lamar Valley. Mutation rates represent rate of mutation per base pair per million years.

We used Bayesian serial coalescent modeling [22], [23] to explore probable effective population sizes. Considering the Lamar Valley (which contained two haplotypes) and the ancient data (which contained a single haplotype), we determined that Ne was likely to be between 5000 and 10000 individuals (Fig. 2), and that the population has remained at a relatively constant size through time.

## Discussion

The low mitochondrial diversity that we observed in modern *A. t. melanostictum* is comparable to the low mtDNA diversity described in other *Ambystoma* populations [26], [27], and consistent with the low nuclear microsatellite diversity described for this population [28].

H1 shares more than 99% identity with mitochondrial sequences of *A. t. melanostictum* collected from other geographic regions in the Great Plains and the Pacific Northwest [24]. All of the Yellowstone haplotypes showed the greatest similarity to *A. t. melanostictum* collected from the eastern Great Plains. The greater Yellowstone ecosystem was covered by ice until ∼14,000 ybp, and the region was colonized by amphibians soon after this ice cap melted, and this genetic similarity is consistent with a recolonization westward from the Great Plains. The low diversity throughout the region suggests that there has been relatively little gene exchange with outside populations since this initial colonization, however the mutation rate and overall diversity is so low that moderate levels of migration and gene exchange cannot be excluded, especially from populations with identical haplotypes present. Although diversity was low, the distribution of the four haplotypes revealed significant spatial differentiation (Fig. 1). We found significant isolation-by-distance as well as significant population subdivision (Table 2). The amount of spatial partitioning even with such low genetic diversity suggests that migration between ponds is low and that philopatry is high.

Our analysis of aDNA indicated that H1 was historically prevalent in this population as well; indeed H1 was the only haplotype identified in the ancient samples. Rodríguez and colleagues [29] recently found a similar lack of mitochondrial variation in 19 ancient specimens of the Iberian linx. Based on previous analyses of strontium isotopes, Lamar Cave specimens are known to originate within a 7-km radius of the cave site [10]. We therefore used the ponds in Lamar Valley (Fig. 1 inset), all within 7 km of the cave, as the modern descendants of the population represented in Lamar Cave. Within the modern Lamar Valley population, 71 of 74 (96%) samples represented H1. If haplotype frequencies had remained entirely consistent over the past several millennia, the chance of independently sampling H1 in all of the 16 samples would be 0.96^16^ = 0.52 or 52%. This relatively large probability suggests that the spatial distribution of modern haplotypes reflects patterns that have likely persisted for millennia, and that the haplotype distribution seen in the current Lamar Valley is similar to the distribution that has existed for several thousand years.

Of course, haplotype frequencies do change through time and even if populations remain geographically stable, new haplotypes are introduced by mutation. To construct models representing the dynamic nature of population genetics, we simulated coalescence based on a range of population sizes and mutation rates [22]. The mitochondrial mutation rate in *Ambystoma* may be as high as 1.5% mutations per base pair per million years in the D-loop, but is probably closer to 0.75% in coding regions such as the cytochrome-b gene [24], [30]. The region sequenced spans coding regions, an intergenic spacer region and the D-loop, the mutation rate is certainly not consistent across all sequenced base pairs. Indeed, we did not observe any polymorphisms in any of the sequenced coding region. We used a relaxed Bayesian molecular clock to explore a range of mutation rates, and present the upper (1.75%) and lower (0.25%) expected limits of mutation rates. We calculated Tajima's D for the Lamar Valley population (as well for the entire population) to be indistinguishable from zero, and we thus used a constant population size for all of our models. The effective population size of Lamar Valley *A. t. melanostictum* was simulated using the number of mitochondrial segregating sites identified in ancient (Fig. 2A) or identified in both modern and ancient (Fig. 2B) to evaluate the success of each simulation. When we considered only the ancient individuals and segregating sites, we found a small population to be maximally likely (Fig. 2A). Considering both modern and ancient individuals and segregating sites, we estimated the effective population size to be between 3000 and 10000 (Fig. 2B). We describe a population that has experienced little genetic change through time and shows no variation in aDNA, reminiscent of the situation seen in some mammalian populations [29].

Base pair misincorporations, specifically cytosine-deamination transitions are shown to be pervasive in amplifications of ancient and historic DNA, even from samples as young as 50 years [31], [32], [33] showed that base pair misincorporations, specifically cytosine deamination-mediated transitions, are pervasive in amplifications. However, in this study, we genotyped 16 *A. t. melanostictum* ranging in age from 100 to 3300 years, sequencing a total of more than 10,000 ancient base pairs with a minimum of 2× coverage. PCR misincorporations were not detected in any of the sequences, showing that DNA from all these samples could be faithfully amplified. While our laboratory has performed numerous aDNA-based studies (i.e. [12], [13], [17], [34], [35]), all these studies were based on mammalian DNA, and amphibian material had never been handled in our laboratory. We performed all the work with ancient amphibian DNA before any of the modern DNA or tissue entered our laboratory, and indeed before the field work was performed, making it highly unlikely that the ancient haplotype we isolated was the result of contamination with modern genetic material.

The vertebrate specimens used in the present study, like other samples from Lamar Cave [12], [13], [15], [36] were consumed by raptors or mammal predators, regurgitated or defecated, and then aged in cave conditions [9], [37], [38]. In contrast, ancient DNA that shows frequent cytosine deamination (in studies such as [39]) originates from bones and teeth of large mammals (wolf, mammoth, bear). The specimens shown to be prone to deamination were not digested as were our samples, but rather decomposed, probably in exposed environments. It is possible that digestion and cave preservation in cool temperate regions are less detrimental to genetic fidelity than the process of exposed decomposition. DNA sequences extracted from late-Holocene specimens often contain lesions that result in misincorporations during PCR. However, this fact should not be extrapolated to mean that every sequence derived from material older than 50 years is unreliable. The conditions under which a sample is preserved appear to play a key role in DNA viability, and sequencing coverage as well as the physical history of a specimen (i.e. its taphonomy) should be considered in assessing credibility of ancient sequence. Indeed, studies of Neanderthal DNA indicate that the temperature and conditions under which samples aged is more important to DNA preservation than age [40], [41].

Signals of population bottlenecks are evident across northern Yellowstone, and these have been attributed to the bottlenecks induced by recent events such as fish stocking in the 1950s [5]. Observing only the modern lack of mitochondrial diversity in the lower Lamar Valley, combined with the genetic signals of bottlenecking and modern evidence of population decline, we would reasonably assume that diversity had recently been lost through a discrete population decline event. Using subfossils from the same area, we describe a population that has undergone little to no genetic change over the last several thousand years. Thus, the additional perspective of ancient genetics suggests that this population has been maintained at a relatively constant size for several millennia. Though they may indeed have been detrimental to local *A. t. melanostictum* population sizes, we conclude that the bottlenecks caused by fish stocking and disease are not the primary cause of the low mitochondrial diversity in this region.
